# Nonthermal laser ablation of high-efficiency semitransparent and aesthetic perovskite solar cells

**DOI:** 10.1515/nanoph-2021-0683

**Published:** 2022-02-10

**Authors:** Junjie Zhao, Nianyao Chai, Xiangyu Chen, Yunfan Yue, Yi-Bing Cheng, Jianrong Qiu, Xuewen Wang

**Affiliations:** State Key Laboratory of Advanced Technology for Materials Synthesis and Processing, Wuhan University of Technology, Wuhan 430070, China; Foshan Xianhu Laboratory of the Advanced Energy Science and Technology, Guangdong Laboratory, Foshan 528216, China; State Key Laboratory of Modern Optical Instrumentation, College of Optical Science and Engineering, Zhejiang University, Hangzhou 310027, China

**Keywords:** non-thermal laser ablation, perovskite solar cells, semi-transparent

## Abstract

Perovskite solar cells (PSC) offer a promising solution for building integrated photovoltaics (BIPVs) due to its high photoelectric conversion efficiency (PCE). However, increasing the transparency of their functional layers dramatically decreases the PCE. Here, a computer controlled laser patterning method was proposed to directly turn PSC modules into semitransparent and with aesthetic artificial pattern, without additional complexities to the conventional PSCs fabrication process. A structured ST-PSC achieving a champion PCE of 17.5% with average visible transparency (AVT) of 18.2%, and a mini-module with 5 × 5 cm^2^ delivering a PCE of 9.1% with AVT of 37.7% were demonstrated. Rationally designed aesthetic patterns were imprinted on mini-modules, achieving a PCE of 14.4%. These results reveal a new route for low-cost facile fabricating high performance large-area aesthetic BIPV modules, and represent a big step forward toward the fabrication of solar cells with high efficiency and high transparency.

## 1 Introduction

Turning cities into power plants is a promising strategy to alleviate the imminent energy crisis [1], [2], [3]. The key point is to find a low-cost and effective way to convert solar energy into electricity in the city area [4]. Integrating transparent photovoltaic devices into buildings is one of the most effective solutions [5]. Due to the attractive opto-electronic properties of perovskite, semi-transparent perovskite solar cells (ST-PSCs) are considered as an excellent candidate providing both shading and green electricity to the building [6]. However, it is contradictory to simultaneously improve the visibility (transparency), PCE and aesthetics of ST-PSCs.

So far, to achieve high AVT of ST-PSCs, the main focus was on the manufacture of transparent perovskite absorber layers and electrodes. A simple strategy is to reduce the thickness of the perovskite film to obtain better visibility [7, 8]. However, as the absorber layer becomes thinner, to achieve a uniform, high surface coverage and pinhole-free thin film becomes a big challenge [9], [10], [11]. Therefore, creating light passing channels with microstructures on the absorber layer is straightforward to increase the transparency of PSCs [12], [13], [14], [15]. An ST-PSC with nanopillar perovskite films yielding a maximum PCE of 10.8% with an AVT of 37.4% was achieved [16]. Except making perovskite layer transparent, ST-PSCs cannot be achieved without transparent electrodes. To achieve high performance transparent electrodes, various techniques have been reported, such as sputtering transparent conductive oxides and thin metals [17, 18], conductive polymers [19], and carbon materials [20], [21], [22]. However, unavoidable damage to underneath functional layers based on current techniques leads to poor performance of ST-PSCs [23]. Therefore, finding a strategy to fabricate both high quality transparent perovskite layer and electrodes is critical for achieving high efficient ST-PSCs.

Herein, a non-thermal ablation technique was proposed to directly fabricate ST-PSCs via femtosecond laser direct writing. The ultrafast energy deposition on each functional layer with femtosecond pulses, enabling removal of all materials by suppressing thermal diffusion into a constraint zone. As a result, low loss micromesh structures were designed and imprinted on the whole cell, directly turning all the functional layers into transparency. Under optimal conditions, a record high 17.5% PCE with 18.2% AVT single junction device, and a mini-module with 5 × 5 cm^2^ delivering a PCE of 9.1% with AVT of 37.7% were achieved. In addition, rationally designed aesthetic mini-modules achieving PCE of 14.4% were also demonstrated.

## 2 Results and discussion

**Figure 1:**
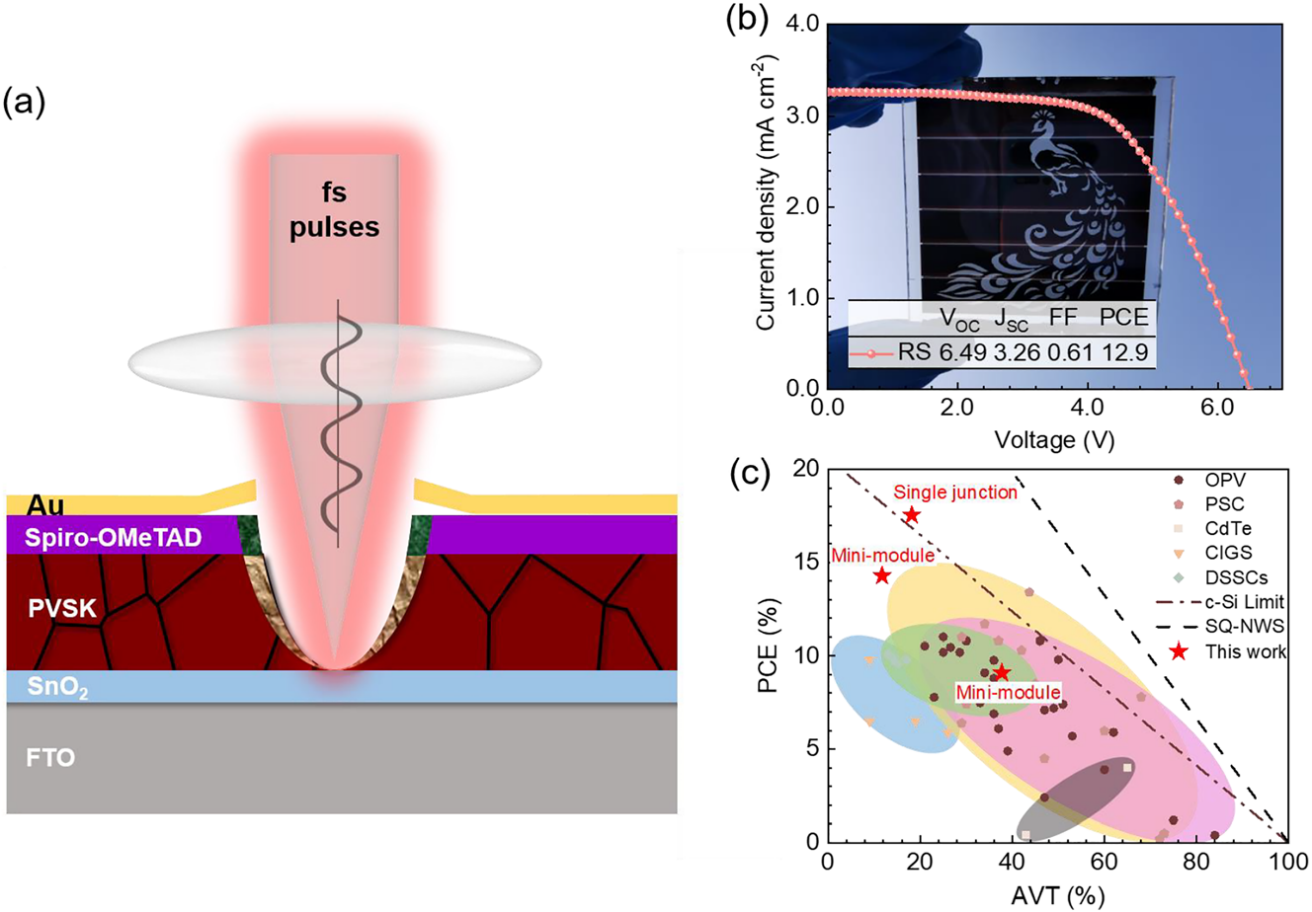
Semi-transparent perovskite solar cells (ST-PSCs) prepared by femtosecond laser direct writing. (a) Schematic of the ablation of a whole PSC cell with all functional layers by a femtosecond laser pulse. (b) Photo and J-V test of a fabricated aesthetic PSC mini-module with 5 × 5 cm^2^ (c) plot of PCE with respect to AVT for transparent and semitransparent PVs reported in the literature. The dash-dot line indicates the theoretical Shockley–Queisser limit for non-wavelength selective PVs. See Table S5 for the detailed reference table.

As depicted in Figure 1, to improve the transparency, micromesh structures were etched through all functional layers in a single step via femtosecond laser direct writing. The light can directly pass through the microstructures. The PSCs obtained by this strategy are semitransparent and artistic, which are suitable for BIPVs. Compared with traditional methods, this approach exhibits a high-efficient and low-cost process which does not require manufacturing thin absorber layer and special transparent electrodes. Most importantly, with computer assisted design, artificially aesthetic patterns can be easily imprinted on a whole PSC module to match the aesthetical design of buildings. A peacock pattern was imprinted on a 5 × 5 cm^2^ mini-module, keeping a PCE of 12.9%. By fabricating micromesh structures on PSC cells, we can obtain both transparent and high efficient ST-PSCs. We compared our results with the other data reported in the literature by plotting the PCEs of traditional and emerging semi-transparent PVs as a function of the corresponding AVTs. The PCEs achieved in this work were the best than those with comparable transparency reported in the collected literature.

**Figure 2:**
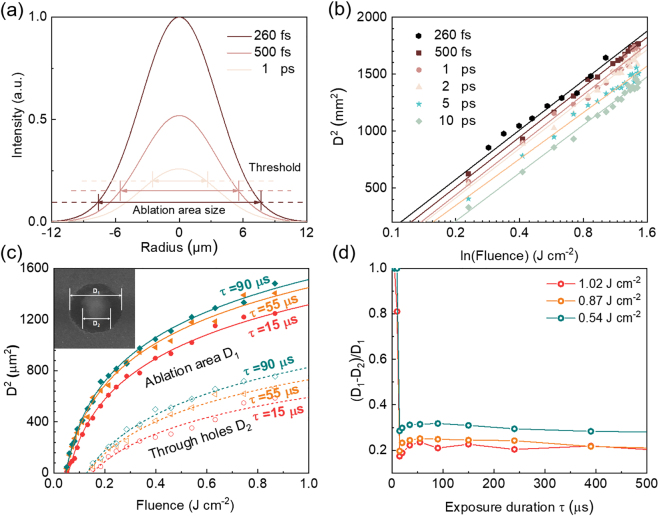
The laser processing parameters of perovskite. (a) The energy intensity distribution of the laser focal plane with different pulse widths. (b) Plot of squared diameter *D*^2^ of ablated area with respect to fluence for pulse widths *d* = 260 fs, 500 fs, 1 ps, 2 ps, 5 ps and 10 ps. (c) Squared diameter *D*^2^ of the ablation area (solid markers) and the through hole (hollow markers) produced in the 550 nm thick perovskite film under exposure duration *τ* = 15 µs, 55 µs, 90 µs (referring to the scanning speed $v=w_{0}/\tau$, 1.67 m/s, 0.45 m/s, 0.28 m/s respectively) irradiation versus applied fluence *F* according to Eq. (1). (d) The relationships between (*D*_1_ − *D*_2_)/*D*_1_ and laser exposure duration under different laser fluence. The pulse duration is 260 fs in (c) and (d).

During the exposure of femtosecond laser pulse, ionization, heating and vaporization do not occur, because the electron-to-ion energy transfer, electron heat conduction and the hydrodynamic takes much longer than the pulse duration, roughly at picosecond time scale [24], [25], [26], [27]. Hence, heat diffusion is frozen during the interaction of the laser pulse with material for picoseconds or shorter laser pulses, which offers greatly reduced thermal damage and heat affected zone (HAZ) on PSCs. Figure S1 shows a stronger decomposition of the perovskite film irradiated by the longer laser pulse in the focusing region at the same pulse energy. For same pulse energy, the peak intensity for longer pulse duration is proportional lower (Figure S1a). Thus, only in a smaller region, the deposited laser fluence reaches the ablation threshold, leading to a smaller ablation crater. Figure 2(a) shows the theoretical size of the ablation zone comparing different pulse durations at the same pulse energy, with laser wavelength 1030 nm and focusing numerical aperture (NA) 0.05. This effect can be observed from the confocal fluorescence images (Figure S1b). The ablation threshold of the perovskite film at different pulse duration was measured by fitting the measured diameters of the ablation crater relatively to the deposition fluence by Eq. (1) [28]:

(1) $$D_{\text{a}blation}^{2}=2\omega_{o}^{2}\left(\ln\;F-\ln\;F_{th}\right)$$

where $\omega_{0}$ refers to the diffraction limited laser focus diameter, $F_{\text{th}}$ stands for the threshold of perovskite. The corresponding fitting curves and measured data are illustrated in Figure 2(b), which demonstrate that the threshold of perovskite is relatively low at short pulse widths, resulting to a large ablation area under shorter pulse laser with same pulse energy. Therefore, lower laser fluence is required to achieve smaller ablation zone, leading to smaller HAZ.

There are two distinct regions in the laser exposure area on the perovskite film as shown in Figure 2(c) inset. One is the HAZ (*D*_1_ − *D*_2_), and the other is the ablated through hole (*D*_2_). The perovskite film is decomposed due to the local temperature arising in the HAZ, as shown in Figure S1b. These two regions are generated by Gaussian-shaped pulses which the energy decays rapidly at the edge (Figure S2). By reducing the deposited laser fluence and exposure time, the size of the HAZ is able to be optimized. The sizes of *D*_1_ and *D*_2_ varying with the laser fluence under different laser exposure duration are shown in Figure 2(c). The decomposition threshold is about 60% lower than the ablation threshold of perovskite. The differences between the HAZ and the ablation hole (*D*_1_ − *D*_2_)/*D*_1_ respecting to the laser exposure duration under different laser fluence are illustrated in Figure 2(d). As the exposure duration increases, the relative size difference increases rapidly and then become steady. In order to improve the fabrication efficiency, a shorter exposure time with less decomposed area is preferred.

**Figure 3:**
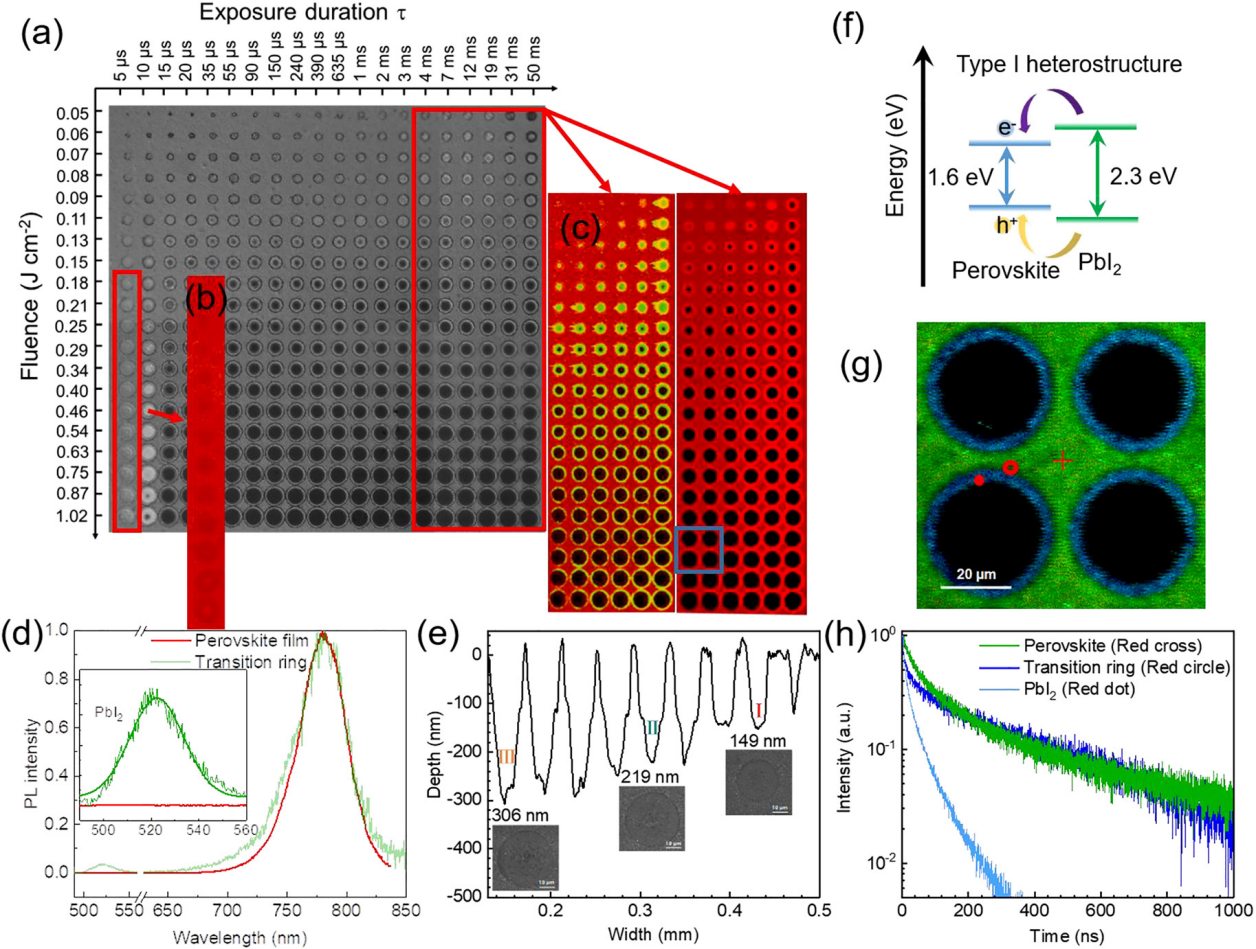
PL and lifetime properties of perovskite films with circular-shaped microholes array. (a) Optical micrograph of perovskite film with circular-shaped micro craters. (b) and (c) Confocal fluorescent images of the typical areas. (d) Photoluminescence (PL) spectra of perovskite films and HAZ, respectively. (e) Stylus profiler image of perovskite film with circular-shaped micro craters. (f) Schematic of the type-I heterostructure for the study of the carrier boundary transportation. Both electrons and holes transfer from PbI_2_ to perovskite. (g) Time-resolved PL (TRPL) mapping of perovskite films. (h) TRPL spectra of selected areas.

The optical micrograph and the corresponding confocal microscope images of an ablation crater array with varying the exposure duration and laser fluence are shown in Figure 3(a)–(c). For organic-inorganic hybrid perovskites, the inorganic components are tightly combined by strong covalent bonds or ionic bonds between atoms, and their lattice deformation will consume a lot of energy. Organic components are bonded together by small molecules through hydrogen bonds or van der Waals, and consume relatively little energy to cause significant lattice deformation [29]. It can be inferred that organic components are more likely to volatilize or decompose under laser irradiation than inorganic components. The photoluminescence spectrum (Figure 3(d)) confirms that the red area is the perovskite film, and the green area is PbI_2_ generated by decomposition of perovskite. More importantly, combining Figure 3(b) and the corresponding thickness change curve obtained from the stylus profiler (Figure 3(e)), it can be verified that as the laser fluence decreases, the thickness of perovskite layer is gradually reduced and no decomposed PbI_2_ is formed in the meantime, which suggests that this process highly suppresses the heat diffusion. The decomposition process leads to a heterojunction formation at the interface of the PbI_2_ (*E*_g_ = 2.3 eV) and the Cs_0.05_[FA_0.85_MA_0.15_]_0.95_Pb(I_0.85_Br_0.15_)_3_ (*E*_g_ = 1.63 eV) (Figure 3(f) and Figure S3) [30]. Both photogenerated electrons and holes diffuse to Cs_0.05_[FA_0.85_MA_0.15_]_0.95_Pb(I_0.85_Br_0.15_)_3_ and recombine there, resulting to an increased fluorescence intensity in the edge of the HAZ (Figure 3(c)). Time-resolved photoluminescence (TRPL) mapping of perovskite and spectra corresponding to different regions (Figure 3(g) and (h)) also illustrate the simultaneous presence of PbI_2_ and perovskite in the HAZ. Such PbI_2_ inclusions can act as a passivation layer for nonradiative defects on the perovskite film [31].

**Figure 4:**
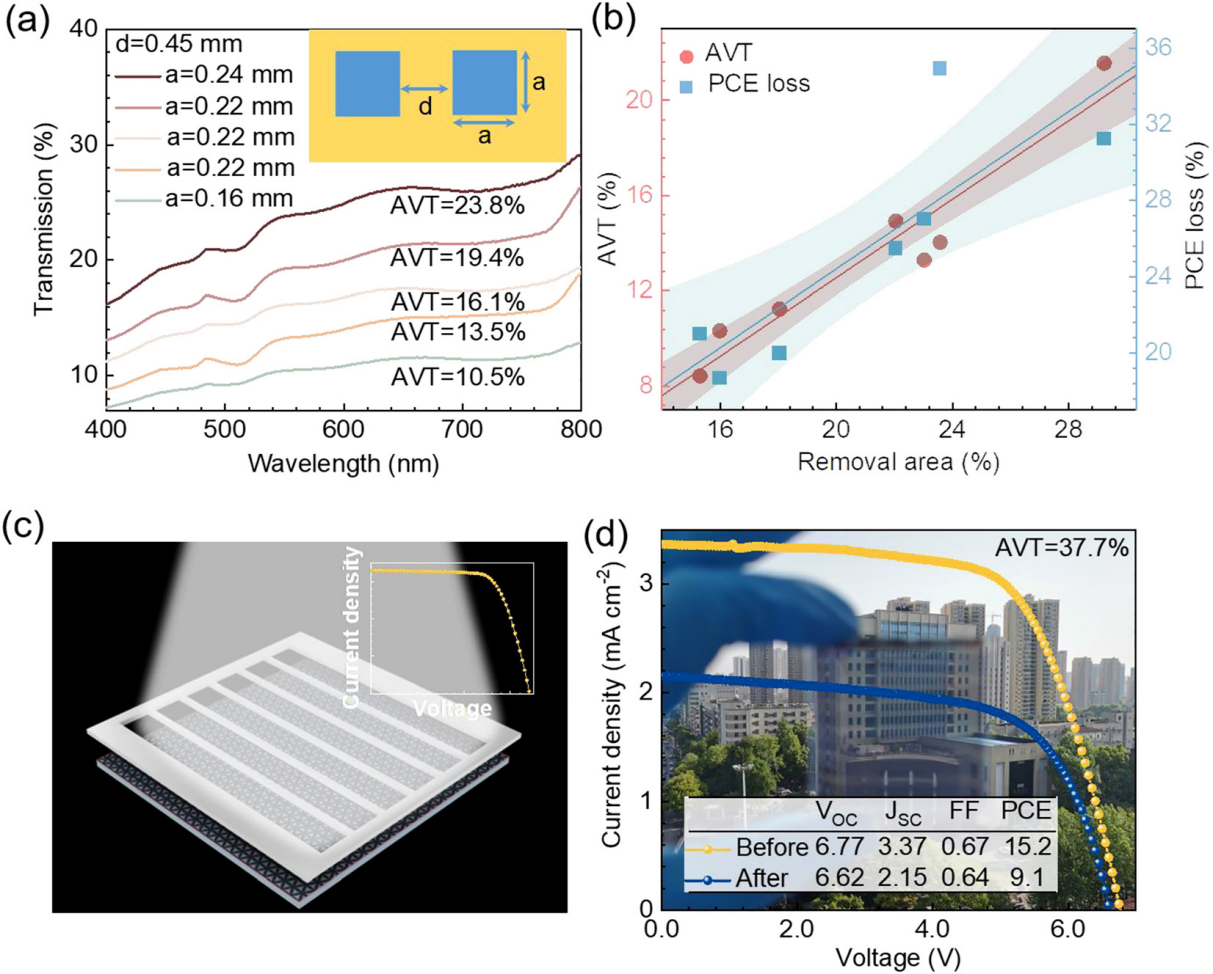
Photovoltaic performance characterizations of ST-PSCs. (a) The transmission curve of ST-PSCs is caused by increasing the side length of the removed square. (b) Transmission and PCE loss are linearly fitted as a function of the removal area. (c) Schematic diagram of mask-assisted PCE test. (d) Comparison of the current−voltage characteristics of 5 × 5 cm^2^ PSCs before and after laser ablation.

For directly ablating whole PSCs, the ablation threshold of the light blocking layers such as the Spiro-MeOTAD layer and Au layer above the perovskite were also investigated. As shown in Figure S4a and b, the ablation thresholds of Spiro-MeOTAD and Au are slightly larger than perovskite, which is beneficial for removing these three materials in one step. The Spiro-MeOTAD and Au located above the perovskite will absorb part of the energy of the laser, so the laser fluence can be appropriately increased to ensure the complete removal of the materials. Besides, the threshold of FTO/ETL (Figure S4c) is much greater than that of other layers, so it can be guaranteed that FTO/ETL will not be damaged during laser processing with a safe fabrication window. The PSCs were prepared through a traditional facile solution based process combined with vacuum evaporation metal electrode. The PSC device fabrication process is depicted in Figure S5a. As shown in Figure S5b, a certain area of the fabricated PSCs is removed by a femtosecond laser, and then an ST-PSCs with a micromesh structure is formed. SEM image and the corresponding confocal fluorescent image (Figure S6) demonstrate that the perovskite layer is almost completely removed, exposing the clean FTO/ETL layer. The removed area has a direct effect on the AVT of ST-PSCs. Figure 4 shows the transmission curve of ST-PSCs caused by increasing the side length of removed square. Obviously, the more removed areas, the greater AVT of the device can be achieved. Figure 4(b) indicates the direct proportional relationship between the removal area rate and AVT. For the simplicity of the test, we adopted a mask-assisted PCE test with an effective area of 17.1 cm^2^ for 6 × 6 cm^2^ mini-module and an effective area of 10 cm^2^ for 5 × 5 cm^2^ mini-module (Figure 4(c)). Finally, we achieved a single-junction ST-PSCs with PCE of 17.5% and AVT of 18.2% (Figure S7a). Furthermore, we achieved large-area 5 × 5 cm^2^ mini-module with PCE of 9.1% and AVT of 37.7% and 6 × 6 cm^2^ mini-module with PCE of 14.1% and AVT of 11.8% (Figure 4(d) and Figure S7b). As shown in Figure 4(d), ST-PSCs fabricated by the femtosecond laser exhibit excellent semitransparent property (See Figure S8 for the detailed AVT curve.).

**Figure 5:**
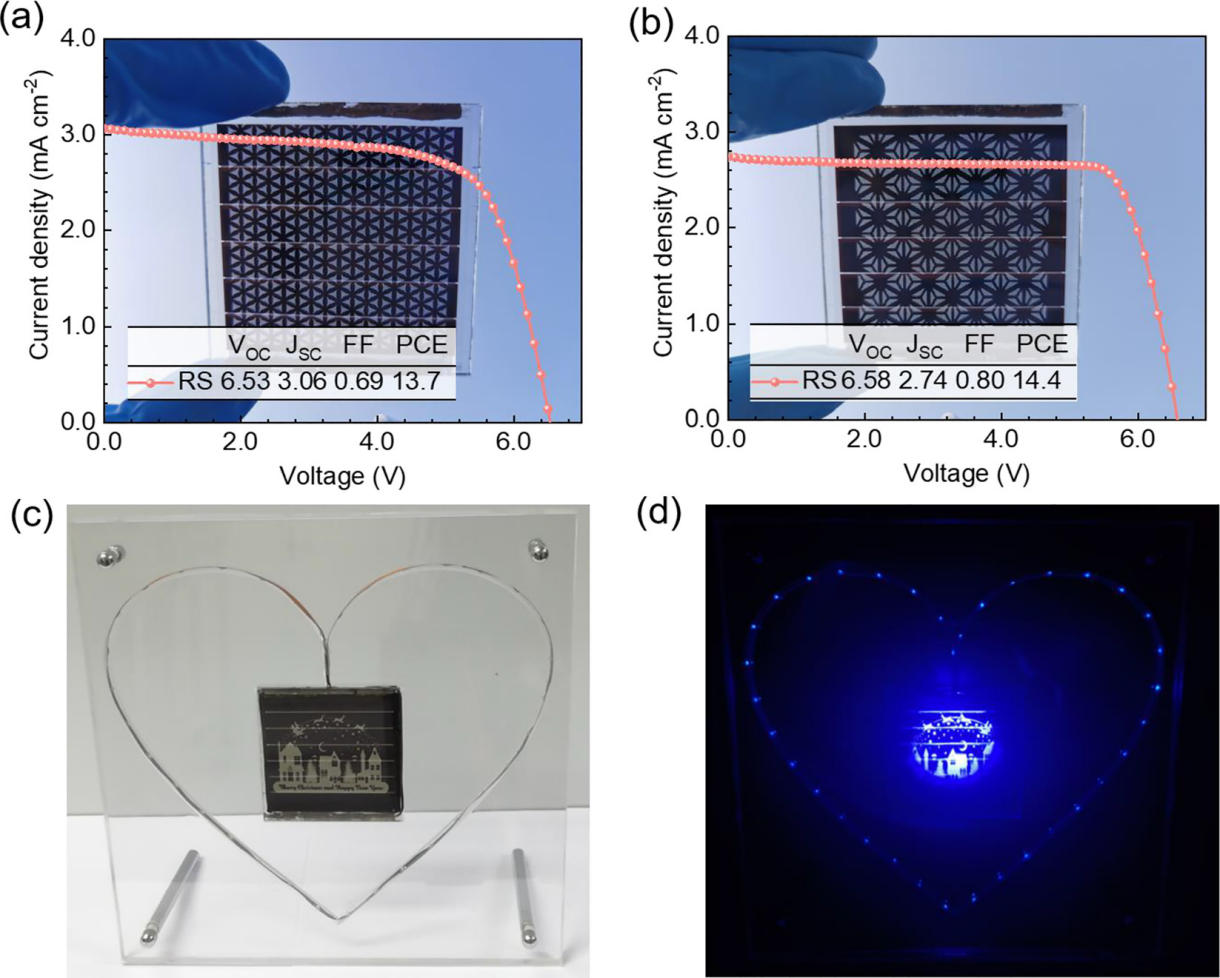
Photovoltaic performance characterizations of rationally designed patterned PSCs. Current−voltage characteristics of PSCs imprinted with (a) origami and (b) symmetrical flowers patterns. (c) and (d) Photo of a PSC Christmas card and lightening more than 35 LEDs under the irradiation of a low intensity dark blue LED lamp.

To further evaluate the role of the proposed ST-PSCs manufacturing method, various rationally designed patterns are imprinted directly on the PSCs. The PSCs imprinted with origami and symmetrical flowers patterns (Figure 5(a) and (b)) can maintain efficiencies of 13.7% and 14.4%, respectively. For more complicated patterns, the PSCs patterned with asymmetric peacock and Chinese characters “Fu” (Figure S9) maintain efficiencies of 12.9% and 12.7%, respectively. This approach offers a totally new route for design and fabricate PSCs. Rational design and broaden applications can be utilized with PSCs for power generation. Figure 5(c) and (d) shows a beautiful designed perovskite solar cell Christmas card fabricated by this technique, which delivers excellent light conversion efficiency even at low light intensity.

## 3 Conclusions

In summary, a computer controlled laser patterning method was proposed to directly turn PSC modules into semitransparent and with aesthetic artificial pattern. This approach is a post-patterning method without additional complexities to the conventional PSCs fabrication process, and enable to tailor the pattern upon the demands from the aesthetic building design. Under the femtosecond laser pulses, the heat effect is highly restrained and localized which enables the removal of metal electrode, hole transport layer and perovskite nearly without thermal decomposition. As a result, a structured ST-PSC achieving a champion PCE of 17.5% with average visible transparency (AVT) of 18.2%, and a mini-module with 5 × 5 cm^2^ delivering a PCE of 9.1% with AVT of 37.7% were demonstrated. Rationally designed aesthetic patterns on mini-modules also achieved a PCE of 14.4%. This strategy makes it promising for manufacturing high performance large-area aesthetic (BIPV) modules by a low-cost and facile route, with architectural appeal of a building by integration at the architectural design stage and being able to added during the initial construction.

## Supplementary Material

Supplementary Material

Supplementary Material
