# Supplementary material for: Nonthermal laser ablation of high-efficiency semitransparent and aesthetic perovskite solar cells
Source: Nanophotonics. 2022 Feb 10;11(5):987–93. doi: 10.1515/nanoph-2021-0683 (PMC11501482; doi:10.1515/nanoph-2021-0683)
Supplement: Supplementary file 2 — Supplementary Material [file j_nanoph-2021-0683_suppl_002.docx]

**Supplementary material**

**Non-thermal laser ablation of high-efficiency semi-transparent and aesthetic perovskite solar cells**

Junjie Zhao ^a,#^, Nianyao Chai ^a,#^, Xiangyu Chen ^a^, Yunfan Yue ^a^, Yi-Bing Cheng ^a,b^, Jianrong Qiu^c^, Xuewen Wang^a,b,*^

^a^ State Key Laboratory of Advanced Technology for Materials Synthesis and Processing, Wuhan University of Technology, Wuhan 430070, PR China;

^b^ Foshan Xianhu Laboratory of the Advanced Energy Science and Technology, Guangdong Laboratory, Foshan 528216, PR China;

^c^ State Key Laboratory of Modern Optical Instrumentation, College of Optical Science and Engineering, Zhejiang University, Hangzhou, 310027 China

**Corresponding Author**

* E-mail address: [xwwang@whut.edu.cn](mailto:xwwang@whut.edu.cn) (XW. Wang)

**Contents**

**1.** **Experimental methods**

**2. Supplementary Figures**

**Figure S1.** The effect of pulse duration on laser processing. (a) When the pulse energy is constant, the light intensity is distributed in the time domain under different pulse duration 260 fs, 500 fs, 1 ps and 2 ps. (b) SEM and confocal fluorescence mapping of perovskite film with circular-shaped microholes.

**Figure S2.** Schematic diagram of short-exposure and long-exposure laser modified of glass-supported Cs_0.05_[FA_0.85_MA_0.15_]_0.95_Pb(I_0.85_Br_0.15_)_3_ films.

**Figure S3.** Ultraviolet-visible absorption spectra (with inset showing the Tauc analyses) for high bandgap perovskite thin films used in this work.

**Figure S4.** Laser ablation parameters of each functional layer. (a-c) Squared diameter D^2^ of the through hole produced FTO, Spiro-MeOTAD and Au film under exposure duration τ = 15 μs, 55 μs, 90 μs irradiation versus applied fluence F, respectively.

**Figure S5.** Schematic diagram of the manufacturing process of PSCs. (a) Schematic diagram of the manufacturing process of perovskite solar cells based on the solution method. (b) Schematic diagram of laser ablation depth of PSCs.

**Figure S6**. Laser ablation of gold-free PSCs. (a) SEM of laser ablation depth of gold-free PSCs. The inserted image is an enlarged view of the FTO layer. (b) confocal PL mapping of laser ablation depth of gold-free PSCs.

**Figure S7.** J-V test. (a) Comparison of the current−voltage characteristics of single-junction (FAPbI_3_)_0.95_(MAPbBr_3_)_0.05_ PSCs before and after laser ablation. (b) EQE spectra of (FAPbI_3_)_0.95_(MAPbBr_3_)_0.05_ devices. (c) Comparison of the current−voltage characteristics of 6×6 cm^2^ PSCs before and after laser ablation.

**Figure S8.** Ultraviolet-visible Transmission spectra of ST-PSCs with various active area

**Figure S9.** J-V test of PSCs with asymmetric pattern. (a) Current−voltage characteristics of PSCs imprinted with peacock. (b) Current−voltage characteristics of PSCs imprinted with Chinese characters “Fu”.

**Figure S10.** The unencapsualted 5×5 cm^2^ module stability of PSCs and ST-PSC after 500 h storing under ambient condition, respectively.

**3. Supplementary Tables**

**Table S1.** Comparison of PL decay fitting parameters between different measurement positions.

**Table S2.** Comparison of J-V characteristic parameters of single-junction PSCs before and after laser ablation.

**Table S3.** Comparison of J-V characteristic parameters of 5×5 cm^2^ before and after laser ablation.

**Table S4.** Comparison of J-V characteristic parameters of 6×6 cm^2^ before and after laser ablation.

**Table S5.** Reference table for Figure 1.

**1.** **Experimental methods**

**Materials.** Dimethyl sulfoxide (DMSO) and Ethyl acetate (EA) were ordered from Sigma-Aldrich Ltd. SnCl_4_·5H_2_O (99.99%) and CsI (99.99%) were all bought from Aladdin Ltd. Formamidinium iodide (FAI) and Methylammonium bromide (MABr) (99.50%) were purchased from Lumtec Ltd. Spiro-OMeTAD (99.81%) was purchased from Shenzhen Feiming Science and Tech Co, Ltd. SnO_2_ colloid solution (15%) was purchased from Alfa Ltd. PbI_2_ (99.99%) was purchased from TCL Ltd. PbBr_2_ (99.99%) was purchased from Xi’an Polymer Light Technology Corp. Commercially available chemicals were used without further purification. All syntheses were performed in glovebox (filled with high purity N_2_).

**PSCs fabrication.** PSCs were fabricated through a facile solution process with a standard configuration of glass/FTO/compact SnO_2_/perovskite/ Spiro-OMeTAD/Au.[1] FTO substrates were firstly cleaned with soap water, ultrasonically cleaned sequentially in ethanol and distilled-water for 30min, respectively. FTO substrates was blown dry with air flow. 1.1 g SnCl_2_·2H_2_O, 5 g urea, 5 mL concentrated hydrochloric acid, 100 μL thioglycolic acid were all dissolved in 400 mL distilled water. The solution was shaken for 10 minutes and then put in the refrigerator to aging for 12 h. After UV treatment for 15 minutes, FTO substrates is immersed in the configured SnCl_2_ precursor solution. FTO substrate is heated in the oven for 3h, then cleaned with deionized water and dried with flowing air. The as-deposition compact SnO_2_ film was sintered at 170 ℃ on hot plate for 60 minutes in air. For the preparation of (FAPbI_3_)_0.95_(MAPbBr_3_)_0.05_ perovskite precursor solution, 7.53 mg MABr, 25.6 mg PbBr_2_, 705.84 mg PbI_2_, 33.76 mg MACl and 240.76 mg FAI were all dissolved in 1000 μL mixed solvents of anhydrous DMF and DMSO (volume ratio, 8:1). For the preparation of Cs_0.05_[FA_0.85_MA_0.15_]_0.95_Pb(I_0.85_Br_0.15_)_3_ perovskite precursor solution, 21.84 mg MABr, 77.07 mg PbBr_2_, 548.6 mg PbI_2_ and 190.06 mg FAI were all dissolved in 800 μL mixed solvents of anhydrous DMF and DMSO (volume ratio, 4:1). 17.3 mg CsI was dissolved and 30 μL DMSO. The latter clear solution is poured into the former clear solution before shake by hand. Shaking is stopped after combining the component solutions. For the preparation of Cs_0.05_[FA_0.85_MA_0.15_]_0.95_Pb(I_0.85_Br_0.15_)_3_ perovskite films, the perovskite was spin-coated on the compact SnO_2_ films at 5000rpm for 30s in the high purity N_2_ glovebox. 100 μL anti-solvent ethyl acetate(EA) is spin-coated on the as-obtained film in order to crystallize and nucleate the perovskite instantaneously. The as-deposited perovskite film was annealed on a hotplate in the glovebox at 120 ℃ for 45 minutes n. After the thermal annealing processes, a Spiro-OMeTAD solution was prepared by adequately dissolving 73 mg Spiro-OMeTAD and 18 μL Li-TFSI (from 520 mg/mL stock acetonitrile solution) and 29 μL FK209 (300 mg/mL stock acetonitrile solution) and 30 μL 4-tert-butylpyridine in 1 mL chlorobenzene (CBZ), then spin-coated on the perovskite films at 5000 rpm for 30 s in the glovebox. Finally, Au electrodes of ~80 nm was deposited on top through a shadow mask by evaporation at a pressure of ~2.5x10^-4^ Pa.

**ST-PSCs fabrication.** PSCs are ablated by a home-built femtosecond laser direct writing system, integrated with a 10 W amplified femtosecond laser source, three-axis controlling sample stages and a galvo scaning system. The laser wavelength is 1030 nm, repetition rate is tunable from single pulse to 1 MHz, and pulse duration is tunable from 10 ps to 260 fs. The laser beam is scanned by the galvo scaning system and focused by a scan lens with an entrance pupil ($D$) and the focal length measured at 5 mm and 100 mm respectively. The scanning speed of the laser beam over the focal plane can be up to 25 m/s. Various and attractive patterns are formed by moving the computer controlled sample stages or scanning the laser beam in the focal plane. Square micromesh structures or artificial pattern are directly ablated on the PSCs.

**PSCs and ST-PSCs characterization.** The surface morphologies of ST-PSCs in ablation areas were all investigated using a metallographic microscope (Olympus, BX51). The depth of laser ablation of ST-PSCs and the film thickness of each functional layer were measured by Stylus Profilers (Bruker, Dektak XT). The J-V curves of the PSCs and ST-PSCs were measured with a Keithley 2400 source meter and a solar simulator under standard 1-sun light intensity illumination of AM 1.5 G (100 mW cm^-2^) (Oriel 94023A, 300 W). The standard intensity of light was calibrated using a standard Si reference solar cell (Oriel, VLSI standards). Small areas PSCs and ST-PSCs was tested using a metal mask of 0.16 cm^2^ with a scan rate of 10 mV s^-1^. Transmittance of perovskite films with different ablation areas under visible light (400 nm-800 nm) were measured by a UV-vis spectrometer (lamda 750S, PerkinElmer) according to equation. The calculation equation of average visible transmission (AVT) under visible light (400 nm-800 nm) is as follows.

$$AVT=\frac{\int_{400}^{800} T(\lambda)d\lambda}{400} (1)$$

where T(λ) is the transmission at a specific wavelength

**Photoluminescence.** Confocal fluorescent scanning inverted microscope (IX83-FV3000, Olympus) equipped with an Airyscan module was used to map the local PL signal of perovskite films in the vicinity of their laser-processed areas and openings at diffraction-limited resolution. Perovskite film with circular-shaped microholes (green fluorescence, Ex: 405 nm, Em: 450−600 nm; red fluorescence, Ex: 405 nm, Em: 600−800 nm). Steady-state PL measurements were undertaken on a homemade system using a 405-nm laser excitation source (CrystaLaser, Model BLC-050-405). Time-resolved PL decay traces were recorded with a Micro Time 200 (Picoquant) confocal microscope using the time-correlated single photon counting technique with 405-nm laser excitation at a repetition rate of 1 MHz and detection through a 425-nm long pass filter. The excitation power density was around 400 mW cm^–2^. All PL measurements were conducted at room temperature.

**Biexponential fitting.** The PL decay traces presented in Fig. 2d were fitted to

Equation:

$$I\left( t \right)=A_{1}\exp\left( -\frac{t}{\tau_{1}} \right)+A_{2}\exp\left( -\frac{t}{\tau_{2}} \right) (2)$$

where I(t) is the time-dependent PL intensity, τ_1_ and τ_2_ are the lifetimes of the fast

and slow decay components, respectively, and A1 and A2 are the corresponding

amplitudes. From this, we define the effective lifetime (τ_eff_) by equation:

$$\tau_{\mathrm{eff}}=\frac{A_{1}\tau_{1}+A_{2}\tau_{2}}{A_{1}+A_{2}} (3)$$

**Calculation of laser processing parameters.** The diffraction limited focal size of the laser beam $\omega_{o}$ can be calculated by

$$\omega_{o}=\frac{1.22\lambda}{NA} (4)$$

Where $\lambda$ is the laser wavelength 1030 nm and NA is the numerical aperture determined by the scan lens, with the diameter of the entrance pupil ($D$) and the focal length ($f$) .

$$NA=\frac{D}{f} (5)$$

In this experiment, the entrance pupil and focal length were measured at $D$ =5 mm and $f$ =100 mm, leading to the $NA$ 0.05. Thus, the focus diameter $\omega_{o}$ is 25 μm.

Based on the calculated focal size, the peak power ($P_{\mathrm{peak}}$) and deposited laser fluence ($F$) in the focus region can be calculated. The laser power was carefully measured by an optical power meter.

$$P_{\mathrm{peak}}=\frac{P}{\nu\tau_{d}} (6)$$

Where ν is the laser repetition frequency, $\tau_{d}$ is the laser pulse width.

Laser energy density is defined as energy per unit area, also known as laser fluence.

$$F=\frac{P}{\pi\omega_{o}^{2}\nu} (7)$$

The pulse energy (E) of the laser is calculated by

$$E=\frac{P}{\nu} (8)$$

**2. Supplementary Figure**


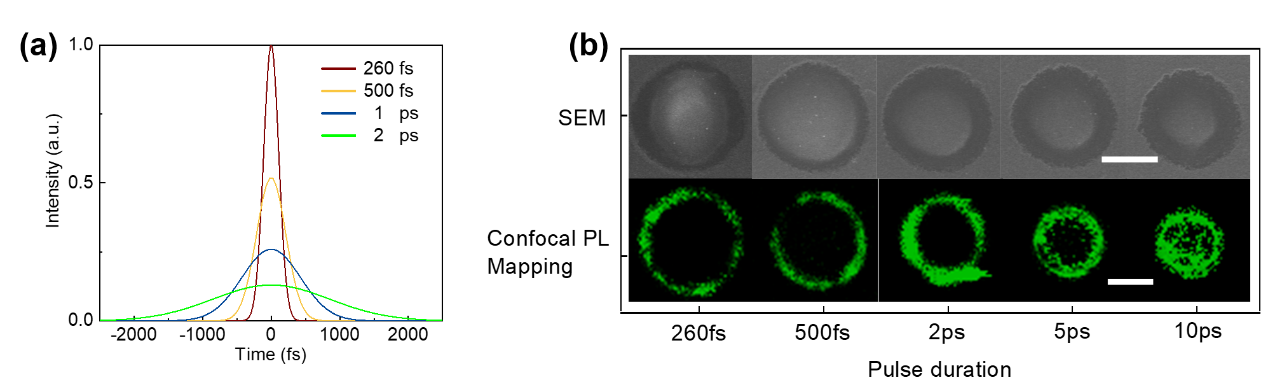


**Figure S1.** The effect of pulse duration on laser processing. (a) When the pulse energy is constant, the light intensity is distributed in the time domain under different pulse duration 260 fs, 500 fs, 1 ps and 2 ps. (b) SEM and confocal fluorescence mapping of perovskite film with circular-shaped microholes.


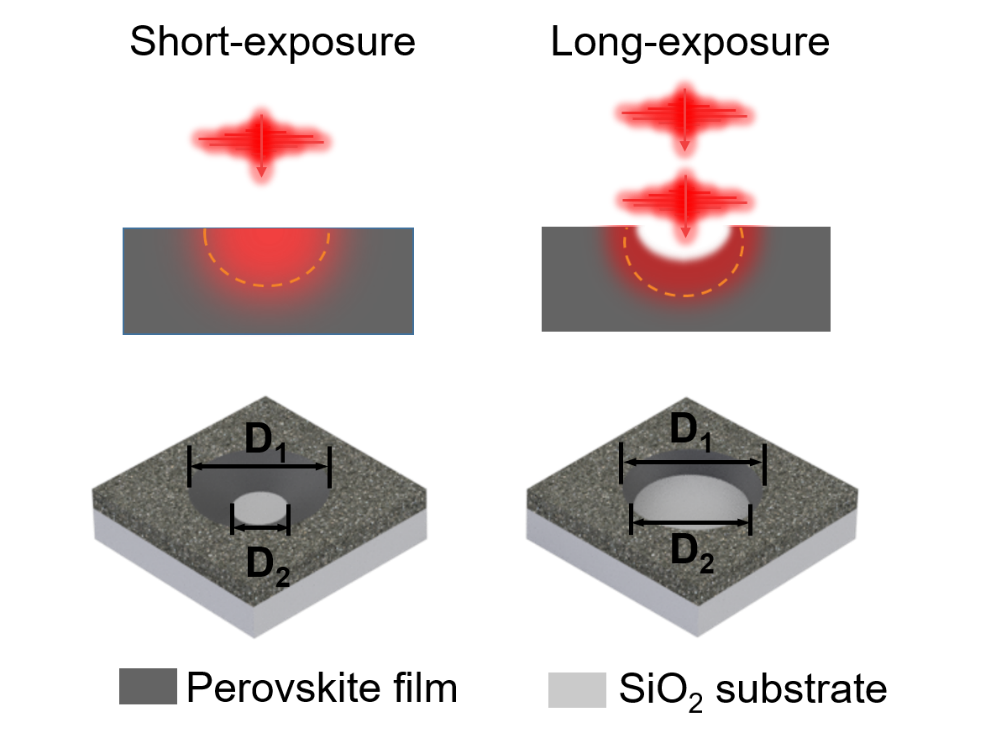


**Figure S2.** Schematic diagram of short-exposure and long-exposure laser modified of sodium-calcium glass-supported Cs_0.05_[FA_0.85_MA_0.15_]_0.95_Pb(I_0.85_Br_0.15_)_3_ films.


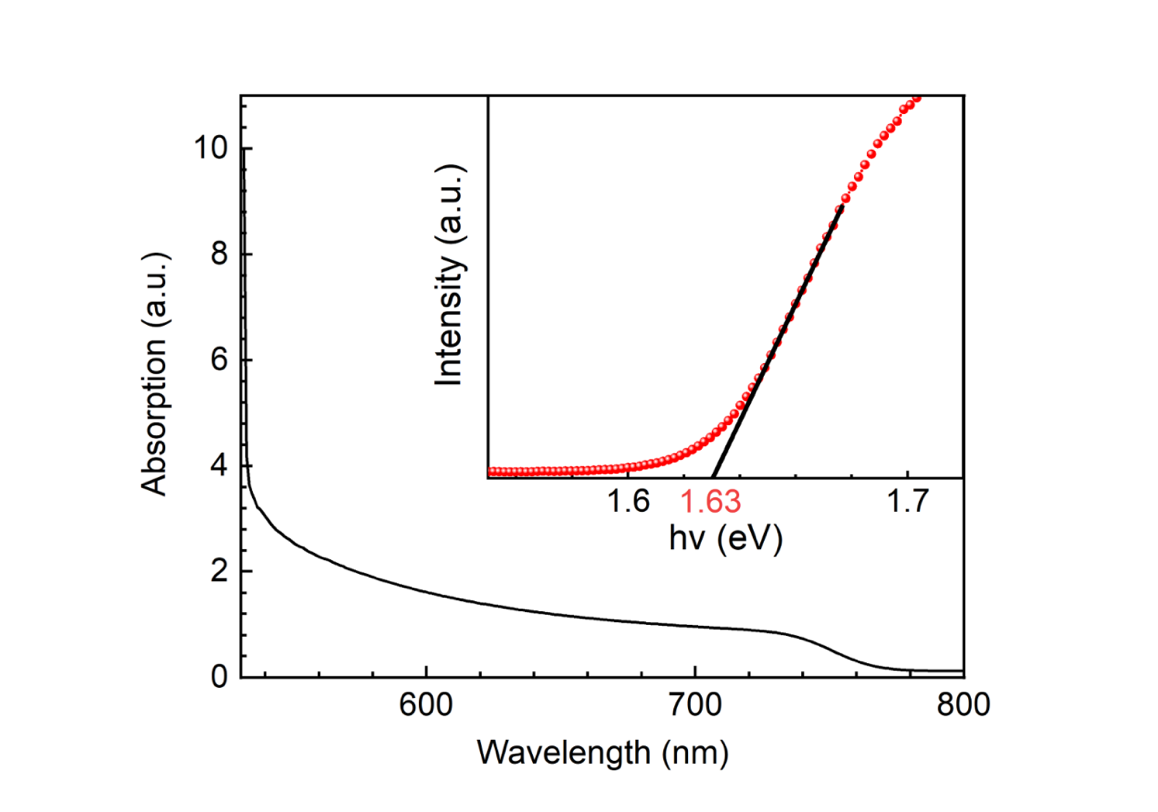


**Figure S3.** Ultraviolet-visible absorption spectra (with inset showing the Tauc analyses) for high bandgap perovskite thin films used in this work.


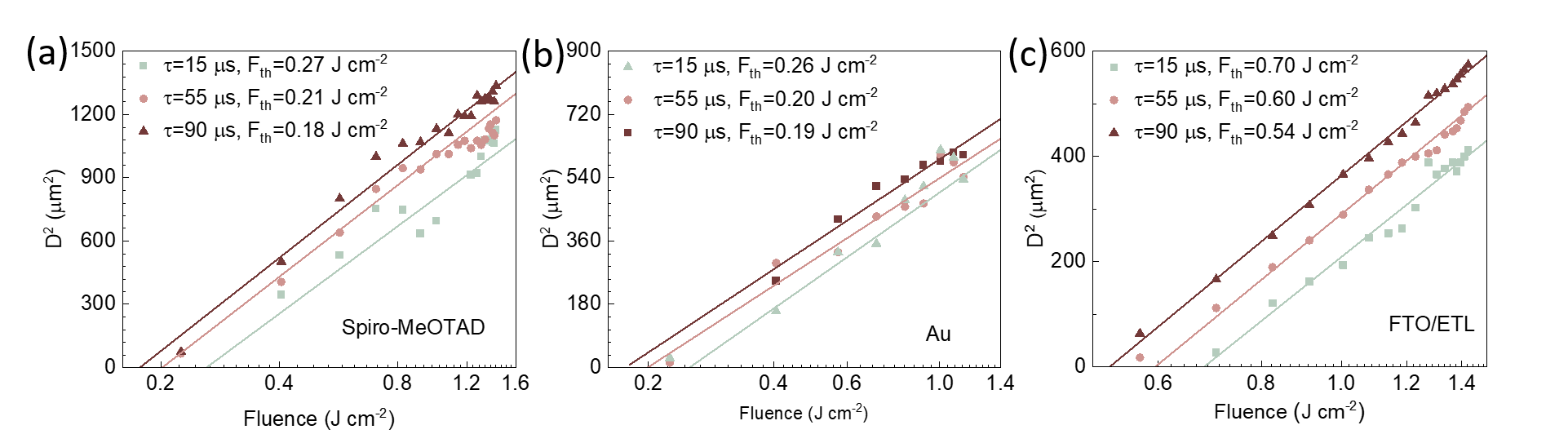


**Figure S4.** Laser ablation parameters of each functional layer. (a-c) Squared diameter D^2^ of the through hole produced FTO, Spiro-MeOTAD and Au film under exposure duration τ = 15 μs, 55 μs, 90 μs irradiation versus applied fluence F, respectively.


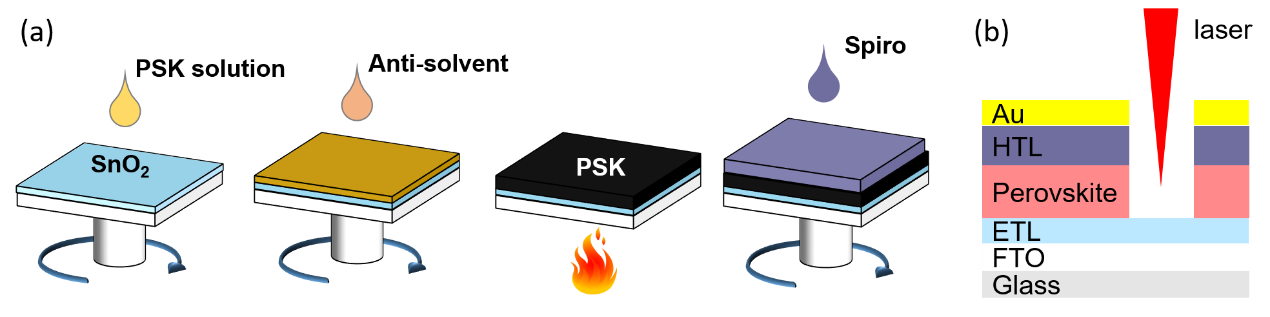


**Figure S5.** Schematic diagram of the manufacturing process of PSCs. (a) Schematic diagram of the manufacturing process of perovskite solar cells based on the solution method. (b) Schematic diagram of laser ablation depth of PSCs.


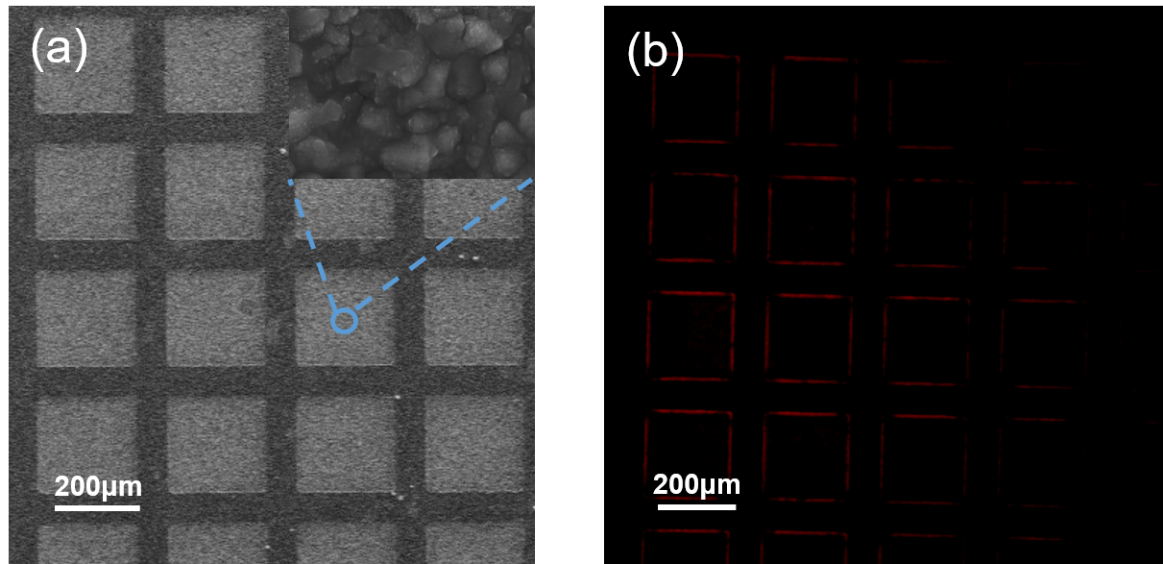


**Figure S6**. Laser ablation of gold-free PSCs. (a) SEM of laser ablation depth of gold-free PSCs. The inserted image is an enlarged view of the FTO layer. (b) confocal PL mapping of laser ablation depth of gold-free PSCs.


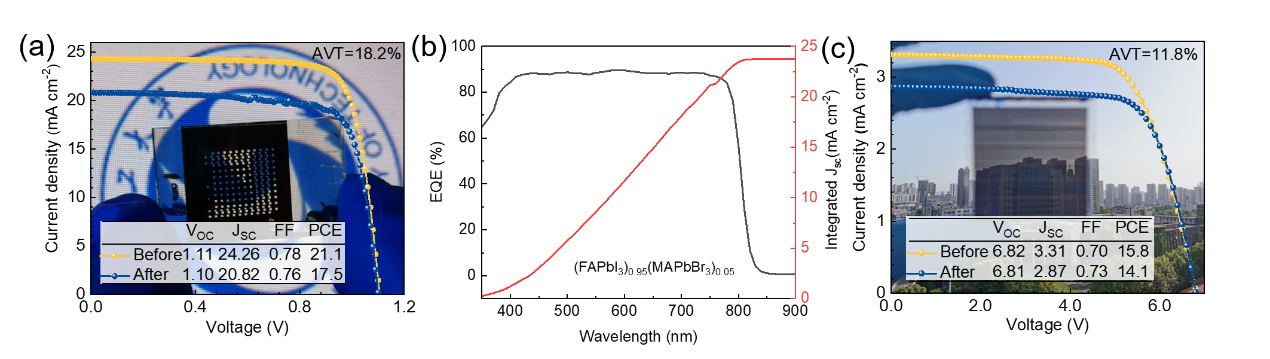


**Figure S7.** J-V test. (a) Comparison of the current−voltage characteristics of single-junction (FAPbI_3_)_0.95_(MAPbBr_3_)_0.05_ PSCs before and after laser ablation. (b) EQE spectra of (FAPbI_3_)_0.95_(MAPbBr_3_)_0.05_ devices. (c) Comparison of the current−voltage characteristics of 6×6 cm^2^ PSCs before and after laser ablation.


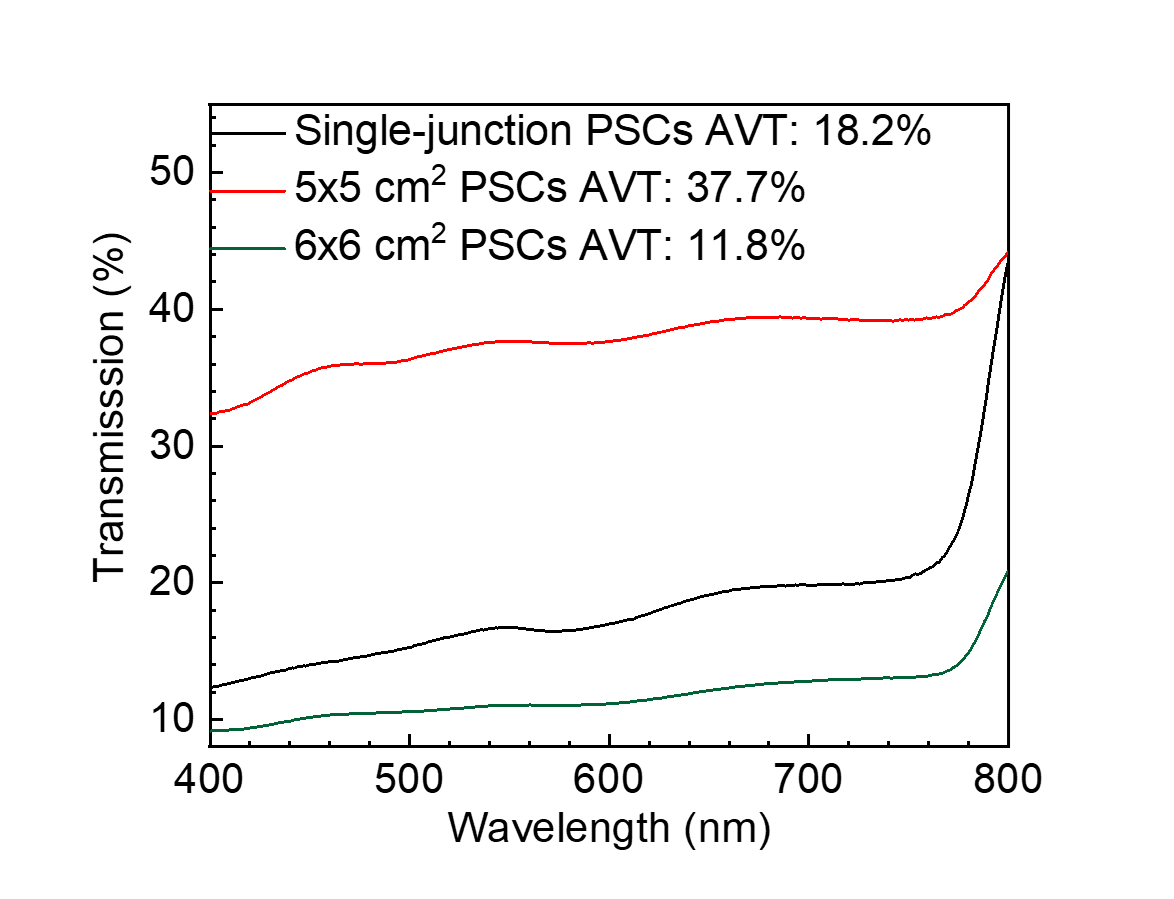


**Figure S8.** Ultraviolet-visible transmission spectra of ST-PSCs with various active areas.


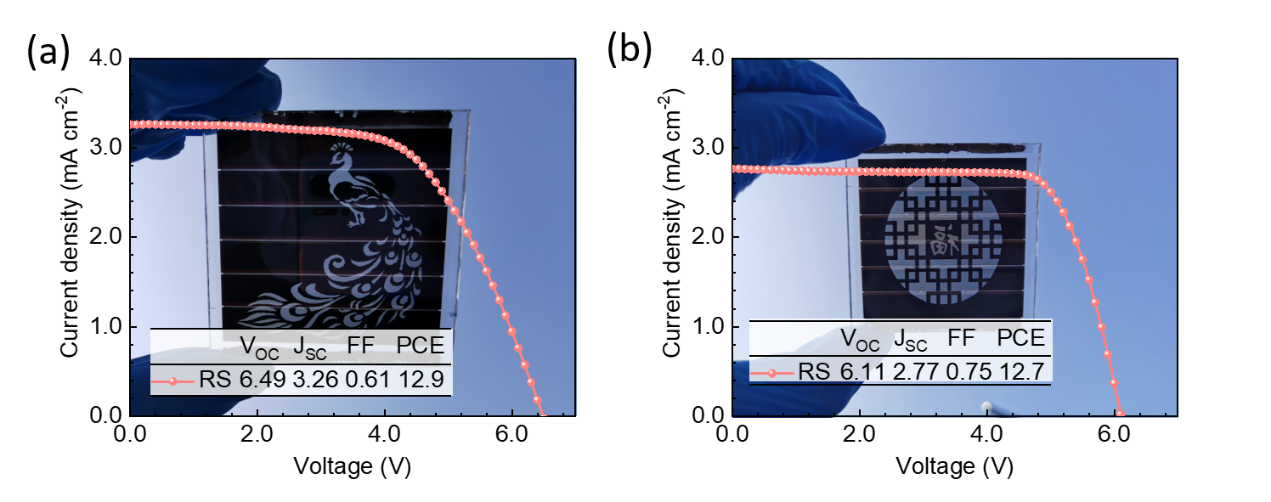


**Figure S9.** J-V test of PSCs with asymmetric pattern. (a) Current−voltage characteristics of PSCs imprinted with peacock. (b) Current−voltage characteristics of PSCs imprinted with Chinese characters “Fu”.


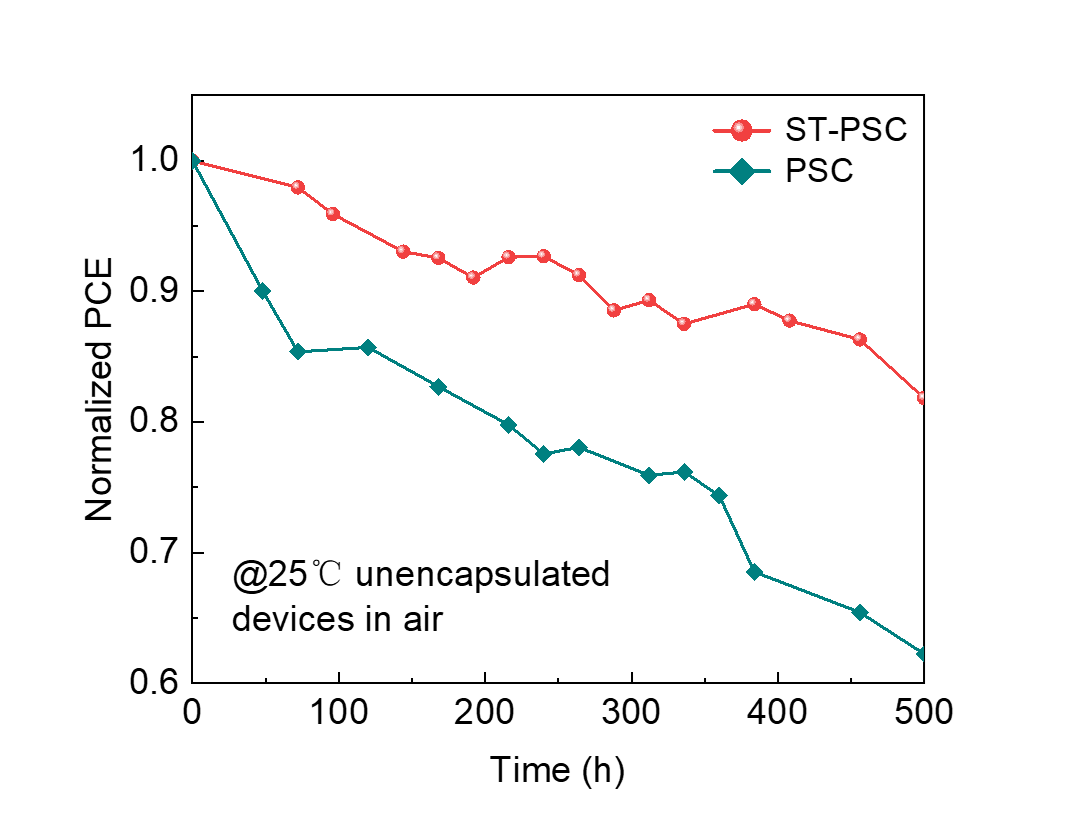


**Figure S10.** The unencapsualted 5×5 cm^2^ module stability of PSCs and ST-PSC after 500 h storage under ambient condition, respectively.

**3.Supplementary Table**

**Table S1.** Comparison of PL decay fitting parameters between different measurement positions.

| Sample | A_1_ | A_2_ | tau_1_ [ns] | tau_2_ [ns] | tau_Av_ [ns] |
| --- | --- | --- | --- | --- | --- |
| PSK | 0.614 | 0.308 | 44 | 321 | 263 |
| HAZ | 0.470 | 0.345 | 17 | 330 | 309 |

**Table S2.** Comparison of J-V characteristic parameters of single-junction PSCs before and after laser ablation.

| Before femtosecond laser ablation | | | After femtosecond laser ablation | | |
| --- | --- | --- | --- | --- | --- |
| Light Intensity | Reverse Scan | Forward Scan | Light Intensity | Reverse Scan | Forward Scan |
| V_oc_ (mV) | 1105 | 1111 | V_oc_ (mV) | 1101 | 1103 |
| J_sc_ (mA/cm^2^) | 24.26 | 24.27 | J_sc_ (mA/cm^2^) | 20.82 | 20.87 |
| Fill Factor | 0.788 | 0.799 | Fill Factor | 0.765 | 0.766 |
| Efficiency (%) | 21.13 | 21.55 | Efficiency (%) | 17.54 | 17.63 |
| V_max_ (V) | 0.94 | 0.95 | V_max_ (V) | 0.93 | 0.93 |
| R_s_ (ohm cm^2^) | 3.44 | 3.04 | R_s_ (ohm cm^2^) | 3.57 | 3.58 |
| R_sh_ (ohm cm^2^) | 2725.62 | 4362.6 | R_sh_ (ohm cm^2^) | 1286.7 | 3596.49 |

**Table S3.** Comparison of J-V characteristic parameters of 5×5 cm^2^ before and after laser ablation.

| Before femtosecond laser ablation | | | After femtosecond laser ablation | | |
| --- | --- | --- | --- | --- | --- |
| Light Intensity | Reverse Scan | Forward Scan | Light Intensity | Reverse Scan | Forward Scan |
| V_oc_ (mV) | 6770 | 6688 | V_oc_ (mV) | 6617 | 6553 |
| J_sc_ (mA/cm^2^) | 3.37 | 3.38 | J_sc_ (mA/cm^2^) | 2.15 | 2.16 |
| Fill Factor | 0.667 | 0.492 | Fill Factor | 0.639 | 0.642 |
| Efficiency (%) | 15.19 | 11.12 | Efficiency (%) | 9.1 | 9.07 |
| V_max_ (V) | 5.1 | 4.1 | V_max_ (V) | 5.2 | 5.2 |
| R_s_ (ohm cm^2^) | 338.15 | 657.71 | R_s_ (ohm cm^2^) | 369.43 | 369.38 |
| R_sh_ (ohm cm^2^) | 44722.72 | 7133.69 | R_sh_ (ohm cm^2^) | 20859.41 | 6668.44 |

**Table S4.** Comparison of J-V characteristic parameters of 6×6 cm^2^ before and after laser ablation.

| Before femtosecond laser ablation | | | After femtosecond laser ablation | | |
| --- | --- | --- | --- | --- | --- |
| Light Intensity | Reverse Scan | Forward Scan | Light Intensity | Reverse Scan | Forward Scan |
| V_oc_ (mV) | 6820 | 6771 | V_oc_ (mV) | 6805 | 6748 |
| J_sc_ (mA/cm^2^) | 3.31 | 3.35 | J_sc_ (mA/cm^2^) | 2.86 | 2.87 |
| Fill Factor | 0.7 | 0.613 | Fill Factor | 0.726 | 0.641 |
| Efficiency (%) | 15.81 | 13.89 | Efficiency (%) | 14.14 | 12.41 |
| V_max_ (V) | 5.2 | 4.8 | V_max_ (V) | 5.4 | 4.9 |
| R_s_ (ohm cm^2^) | 330.73 | 433.37 | R_s_ (ohm cm^2^) | 277.25 | 403.96 |
| R_sh_ (ohm cm^2^) | 53790.5 | 6641.55 | R_sh_ (ohm cm^2^) | 14723.61 | 39032.18 |

**Table S5.** Reference table for Fig. 1.

| Technology | year | Journal | Absorber material | AVT, Spectra region | PCE | Reference |
| --- | --- | --- | --- | --- | --- | --- |
| ST-PSCs | 2015 | Energy Environ. Sci. | MAPbI_3_ | 29%, 400nm-800nm | 6.4% | [2] |
|  | 2016 | Nano Energy | MAPbI_3_ | 47%, 300nm-900nm | 4.5% | [3] |
|  | 2016 | Adv. Energy Mater. | MAPbI_3−x_Cl_x_ | 34%, 300nm-800nm  37%  42%  45% | 11.7%  10.8%  10.3%  8.5% | [4] |
|  | 2017 | Adv. Energy Mater. | MAPbI_3_ | 29%, 400nm-900nm | 11% | [5] |
|  | 2018 | Joule | MAPbCl_2.4_Br_0.6_ | 72%, 300nm-900nm  73% | 0.2%  0.5% | [6] |
|  | 2018 | Adv. Mater. | CsPbI_3_ | 60%, 530nm-800nm | 5.98% | [7] |
|  | 2018 | Adv. Optical Mater. | MAPbI_3_ | 43.7%, 500nm-1200nm | 13.4% | [8] |
|  | 2019 | Adv. Mater. | FAPbBr_2.43_Cl_0.57_ | 68%, 380nm-780nm | 7.8% | [9] |
|  | 2020 | Nano Energy | Cs_0.05_(FA_0.85_MA_0.15_)_0.95_Pb(I_0.85_Br_0.15_)_3_ | 30%, 400nm-800nm | 7.4% | [10] |
| ST-OSC | 2013 | Nanoscale | PSEHTT:ICBA | 36%, 300nm-800nm  37%  39%  47% | 6.9%  6.1%  4.9%  2.4% | [11] |
|  | 2013 | Adv. Energy Mater. | PBMMA:PEMA:(TBA)2Mo6Cl14 | 84%, 400nm-900nm | 0.4% | [12] |
|  | 2014 | Adv. Opt. Mater. | Cy7 | 84%, 400nm-900nm | 0.4% | [13] |
|  | 2017 | Adv. Mater. | PTB7-Th: IHIC | 36%, 370nm-740nm | 9.77% | [14] |
|  | 2019 | joule | PTB7-Th:IEICO-4F | 30%, 380nm-780nm | 10.8% | [15] |
|  | 2019 | Adv. Mater. | PCE‐10:BT‐CIC:TT‐FIC | 36%, 300nm-800nm  49% | 8.8%  7.2% | [16] |
|  | 2019 | Adv. Mater. | PTB7‐Th:6TIC‐4F | 62%, 380nm-780nm | 5.9% | [9] |
|  | 2020 | ACS Appl. Mater. Interfaces | BHJ/MoO3 | 36.2%, 300nm-900nm  28.6% | 8.1%  10.2% | [17] |
|  | 2020 | Sci. Bull. | PTB7-Th:FOIC | 34%, 350nm-950nm  25% | 9.1%  10.2% | [18] |
|  | 2020 | Adv. Energy Mater. | PTB7:FOIC:PC71BM | 50%,300nm-700nm  51% | 9.8%  7.4% | [19] |
|  | 2020 | ACS Appl. Mater. Interfaces | DPP2T:IEICO‐4F | 53%,400nm-600nm  60% | 5.7%  3.9% | [20] |
|  | 2020 | Adv. Opt. Mater. | COi8DFIC | 75%, 400nm-900nm | 1.2% | [21] |
|  | 2020 | Proc. Natl. Acad. Sci. U.S.A. | PCE-10:A078 | 43.3%,380-800nm  25%  46%  47% | 8.1%  11%  10.8%  7.1% | [22] |
|  | 2020 | Solar RRL | PTB7‐Th:IEICO‐4F | 33%, 380nm-780nm  23% | 7.49%  7.79% | [23] |
|  | 2020 | Adv. Energy Mater. | PBDB‐T‐2F:Y6 | 21%, 300nm-850nm | 10.53％ | [24] |
|  | 2020 | Solar Energy | PCE-10 | 26.56%, 300nm-1000nm | 10.46% | [25] |
| DSSCs | 2020 | Nanoscale | SGT‐021 | 13%, 300nm-800nm  14%  15%  17% | 10.1%  9.9%  9.6%  9.8% | [26] |
|  | 2020 | ACS Appl. Energy Mater. | PdTPBP/BPEA | 43%, 400nm-800nm | 7.8% | [27] |
| CdTe | 2013 | Thin Solid Films |  | 65%, 400-1300 nm | 4% | [28] |
|  | 2017 | Thin Solid Films |  | 43%, 400nm-900nm | 0.4% | [29] |
| CIGS | 2016 | Nano Energy |  | 9%, 500nm-1200nm | 6.5% | [30] |
|  | 2016 | J. Mater. Chem. A |  | 26%, 400nm-1000nm | 5.9% | [31] |
|  | 2019 | Solar Energy |  | 9%, 420nm-720nm  19% | 9.8%  6.5% | [32] |

**Reference**

[1] T. Bu, X. Liu, Y. Zhou, J. Yi, X. Huang, L. Luo, J. Xiao, Z. Ku, Y. Peng, F. Huang, Y.-B. Cheng, J. Zhong, A novel quadruple-cation absorber for universal hysteresis elimination for high efficiency and stable perovskite solar cells, Energy Environ. Sci. 10 (2017) 2509–2515. https://doi.org/10.1039/C7EE02634J.

[2] C. Roldán-Carmona, O. Malinkiewicz, R. Betancur, G. Longo, C. Momblona, F. Jaramillo, L. Camacho, H.J. Bolink, High efficiency single-junction semitransparent perovskite solar cells, Energy Env. Sci. 7 (2014) 2968–2973. https://doi.org/10.1039/C4EE01389A.

[3] S. Bag, M.F. Durstock, Efficient semi-transparent planar perovskite solar cells using a ‘molecular glue,’ Nano Energy. 30 (2016) 542–548. https://doi.org/10.1016/j.nanoen.2016.10.044.

[4] H.-C. Kwon, A. Kim, H. Lee, D. Lee, S. Jeong, J. Moon, Parallelized Nanopillar Perovskites for Semitransparent Solar Cells Using an Anodized Aluminum Oxide Scaffold, Adv. Energy Mater. 6 (2016) 1601055. https://doi.org/10.1002/aenm.201601055.

[5] J. Zhao, K.O. Brinkmann, T. Hu, N. Pourdavoud, T. Becker, T. Gahlmann, R. Heiderhoff, A. Polywka, P. Görrn, Y. Chen, B. Cheng, T. Riedl, Self-Encapsulating Thermostable and Air-Resilient Semitransparent Perovskite Solar Cells, Adv. Energy Mater. 7 (2017) 1602599. https://doi.org/10.1002/aenm.201602599.

[6] D. Liu, C. Yang, R.R. Lunt, Halide Perovskites for Selective Ultraviolet-Harvesting Transparent Photovoltaics, Joule. 2 (2018) 1827–1837. https://doi.org/10.1016/j.joule.2018.06.004.

[7] W. Chen, J. Zhang, G. Xu, R. Xue, Y. Li, Y. Zhou, J. Hou, Y. Li, A Semitransparent Inorganic Perovskite Film for Overcoming Ultraviolet Light Instability of Organic Solar Cells and Achieving 14.03% Efficiency, Adv. Mater. 30 (2018) 1800855. https://doi.org/10.1002/adma.201800855.

[8] Z. Ying, W. Chen, Y. Lin, Z. He, T. Chen, Y. Zhu, X. Zhang, X. Yang, A.B. Djurišić, Z. He, Supersmooth Ta _2_ O _5_ /Ag/Polyetherimide Film as the Rear Transparent Electrode for High Performance Semitransparent Perovskite Solar Cells, Adv. Opt. Mater. (2018) 1801409. https://doi.org/10.1002/adom.201801409.

[9] L. Zuo, X. Shi, W. Fu, A.K. ‐Y. Jen, Highly Efficient Semitransparent Solar Cells with Selective Absorption and Tandem Architecture, Adv. Mater. 31 (2019) 1901683. https://doi.org/10.1002/adma.201901683.

[10] J.C. Yu, J. Sun, N. Chandrasekaran, C.J. Dunn, A.S.R. Chesman, J.J. Jasieniak, Semi-transparent perovskite solar cells with a cross-linked hole transport layer, Nano Energy. 71 (2020) 104635. https://doi.org/10.1016/j.nanoen.2020.104635.

[11] W. Jose da Silva, H.P. Kim, Abd. Rashid bin Mohd Yusoff, J. Jang, Transparent flexible organic solar cells with 6.87% efficiency manufactured by an all-solution process, Nanoscale. 5 (2013) 9324. https://doi.org/10.1039/c3nr03011c.

[12] Y. Zhao, R.R. Lunt, Transparent Luminescent Solar Concentrators for Large-Area Solar Windows Enabled by Massive Stokes-Shift Nanocluster Phosphors, Adv. Energy Mater. 3 (2013) 1143–1148. https://doi.org/10.1002/aenm.201300173.

[13] Y. Zhao, G.A. Meek, B.G. Levine, R.R. Lunt, Near-Infrared Harvesting Transparent Luminescent Solar Concentrators, Adv. Opt. Mater. 2 (2014) 606–611. https://doi.org/10.1002/adom.201400103.

[14] W. Wang, C. Yan, T.-K. Lau, J. Wang, K. Liu, Y. Fan, X. Lu, X. Zhan, Fused Hexacyclic Nonfullerene Acceptor with Strong Near-Infrared Absorption for Semitransparent Organic Solar Cells with 9.77% Efficiency, Adv. Mater. 29 (2017) 1701308. https://doi.org/10.1002/adma.201701308.

[15] R. Xia, C.J. Brabec, H.-L. Yip, Y. Cao, High-Throughput Optical Screening for Efficient Semitransparent Organic Solar Cells, Joule. 3 (2019) 2241–2254. https://doi.org/10.1016/j.joule.2019.06.016.

[16] Y. Li, C. Ji, Y. Qu, X. Huang, S. Hou, C. Li, L. Liao, L.J. Guo, S.R. Forrest, Enhanced Light Utilization in Semitransparent Organic Photovoltaics Using an Optical Outcoupling Architecture, Adv. Mater. 31 (2019) 1903173. https://doi.org/10.1002/adma.201903173.

[17] B.-H. Jiang, H.-E. Lee, J.-H. Lu, T.-H. Tsai, T.-S. Shieh, R.-J. Jeng, C.-P. Chen, High-Performance Semitransparent Organic Photovoltaics Featuring a Surface Phase-Matched Transmission-Enhancing Ag/ITO Electrode, ACS Appl. Mater. Interfaces. 12 (2020) 39496–39504. https://doi.org/10.1021/acsami.0c10906.

[18] M. Yao, T. Li, Y. Long, P. Shen, G. Wang, C. Li, J. Liu, W. Guo, Y. Wang, L. Shen, X. Zhan, Color and transparency-switchable semitransparent polymer solar cells towards smart windows, Sci. Bull. 65 (2020) 217–224. https://doi.org/10.1016/j.scib.2019.11.002.

[19] Q. Liu, L.G. Gerling, F. Bernal‐Texca, J. Toudert, T. Li, X. Zhan, J. Martorell, Light Harvesting at Oblique Incidence Decoupled from Transmission in Organic Solar Cells Exhibiting 9.8% Efficiency and 50% Visible Light Transparency, Adv. Energy Mater. 10 (2020) 1904196. https://doi.org/10.1002/aenm.201904196.

[20] J. Lee, H. Cha, H. Yao, J. Hou, Y.-H. Suh, S. Jeong, K. Lee, J.R. Durrant, Toward Visibly Transparent Organic Photovoltaic Cells Based on a Near-Infrared Harvesting Bulk Heterojunction Blend, ACS Appl. Mater. Interfaces. 12 (2020) 32764–32770. https://doi.org/10.1021/acsami.0c08037.

[21] C. Yang, M. Moemeni, M. Bates, W. Sheng, B. Borhan, R.R. Lunt, High‐Performance Near‐Infrared Harvesting Transparent Luminescent Solar Concentrators, Adv. Opt. Mater. 8 (2020) 1901536. https://doi.org/10.1002/adom.201901536.

[22] Y. Li, X. Guo, Z. Peng, B. Qu, H. Yan, H. Ade, M. Zhang, S.R. Forrest, Color-neutral, semitransparent organic photovoltaics for power window applications, Proc. Natl. Acad. Sci. 117 (2020) 21147–21154. https://doi.org/10.1073/pnas.2007799117.

[23] Y. Xiong, R.E. Booth, T. Kim, L. Ye, Y. Liu, Q. Dong, M. Zhang, F. So, Y. Zhu, A. Amassian, B.T. O’Connor, H. Ade, Novel Bimodal Silver Nanowire Network as Top Electrodes for Reproducible and High‐Efficiency Semitransparent Organic Photovoltaics, Sol. RRL. 4 (2020) 2000328. https://doi.org/10.1002/solr.202000328.

[24] W. Song, B. Fanady, R. Peng, L. Hong, L. Wu, W. Zhang, T. Yan, T. Wu, S. Chen, Z. Ge, Foldable Semitransparent Organic Solar Cells for Photovoltaic and Photosynthesis, Adv. Energy Mater. 10 (2020) 2000136. https://doi.org/10.1002/aenm.202000136.

[25] C. Zhu, H. Huang, Z. Jia, F. Cai, J. Li, J. Yuan, L. Meng, H. Peng, Z. Zhang, Y. Zou, Y. Li, Spin-coated 10.46% and blade-coated 9.52% of ternary semitransparent organic solar cells with 26.56% average visible transmittance, Sol. Energy. 204 (2020) 660–666. https://doi.org/10.1016/j.solener.2020.05.027.

[26] M. Aftabuzzaman, C.K. Kim, H. Zhou, H.K. Kim, *In situ* preparation of Ru–N-doped template-free mesoporous carbons as a transparent counter electrode for bifacial dye-sensitized solar cells, Nanoscale. 12 (2020) 1602–1616. https://doi.org/10.1039/C9NR09019C.

[27] K. Kim, S.K. Nam, J.H. Moon, Dual-Band Luminescent Solar Converter-Coupled Dye-Sensitized Solar Cells for High-Performance Semitransparent Photovoltaic Device, ACS Appl. Energy Mater. 3 (2020) 5277–5284. https://doi.org/10.1021/acsaem.0c00171.

[28] C. Heisler, C.S. Schnohr, M. Hädrich, M. Oertel, C. Kraft, U. Reislöhner, H. Metzner, W. Wesch, Transparent CdTe solar cells with a ZnO:Al back contact, Thin Solid Films. 548 (2013) 627–631. https://doi.org/10.1016/j.tsf.2013.09.087.

[29] A. Mutalikdesai, S.K. Ramasesha, Solution process for fabrication of thin film CdS/CdTe photovoltaic cell for building integration, Thin Solid Films. 632 (2017) 73–78. https://doi.org/10.1016/j.tsf.2017.04.036.

[30] K. Kim, W.N. Shafarman, Alternative device structures for CIGS-based solar cells with semi-transparent absorbers, Nano Energy. 30 (2016) 488–493. https://doi.org/10.1016/j.nanoen.2016.10.038.

[31] M. Saifullah, S. Ahn, J. Gwak, S. Ahn, K. Kim, J. Cho, J.H. Park, Y.J. Eo, A. Cho, J.-S. Yoo, J.H. Yun, Development of semitransparent CIGS thin-film solar cells modified with a sulfurized-AgGa layer for building applications, J. Mater. Chem. A. 4 (2016) 10542–10551. https://doi.org/10.1039/C6TA01909A.

[32] M.J. Shin, J.H. Jo, A. Cho, J. Gwak, J.H. Yun, K. Kim, S.K. Ahn, J.H. Park, J. Yoo, I. Jeong, B.-H. Choi, J.-S. Cho, Semi-transparent photovoltaics using ultra-thin Cu(In,Ga)Se2 absorber layers prepared by single-stage co-evaporation, Sol. Energy. 181 (2019) 276–284. https://doi.org/10.1016/j.solener.2019.02.003.
